# MicroRNA-449a Inhibits Tumor Metastasis through AKT/ERK1/2 Inactivation by Targeting Steroid Receptor Coactivator (SRC) in Endometrial Cancer: Erratum

**DOI:** 10.7150/jca.92673

**Published:** 2023-12-02

**Authors:** Yuan Hu, An-Yue Wu, Cong Xu, Ke-Qi Song, Wen-Jing Wang, Xia Yin, Wen Di, Zu-Bei Hong, Li-Hua Qiu

**Affiliations:** 1 Department of Obstetrics and Gynecology, Ren Ji Hospital, School of Medicine, Shanghai JiaoTong University, Shanghai 200127, China; 2 Shanghai Key Laboratory of Gynecologic Oncology, Shanghai 200127, China; 3 State Key Laboratory of Oncogenes and Related Genes, Shanghai Cancer Institute, Ren Ji Hospital, School of Medicine, Shanghai Jiao Tong University

In the previously published version of this article, the representative images of the transwell assays for AN3CA cells transfected with NCs or miR-449a inhibitors in Figure 2F and Figure 4D were inaccurate. The correct images are provided below. This correction will not affect the results and conclusion. The authors sincerely apologize for any inconvenience or misunderstanding that error may have caused.

All authors confirm that they agree to the erratum.

## Figures and Tables

**Figure 2:**
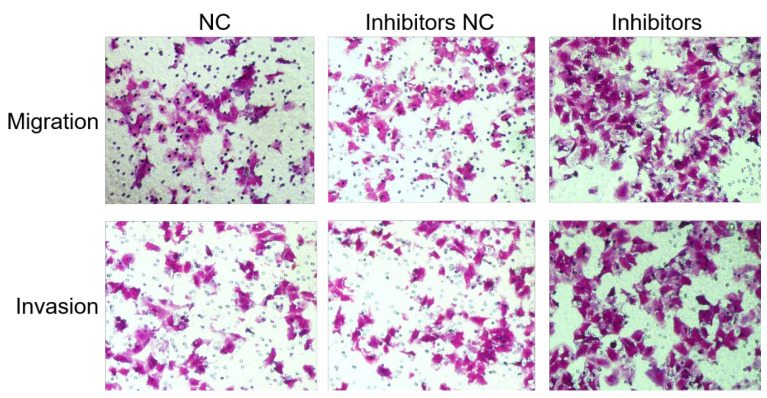
** miR-449a suppresses the migration and invasion of endometrial cancer.** (F) Transwell assay of AN3CA cells transfected with miR-449a inhibitors or NCs. ^*^P < 0.05.

**Figure 4:**
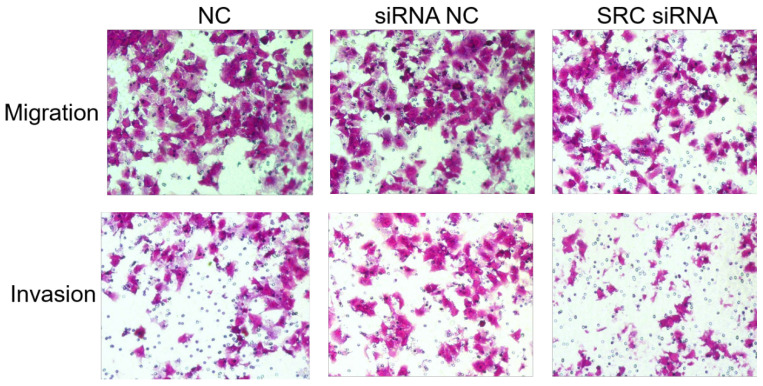
** SRC was upregulated in advanced endometrial cancer tissues and affected migration and invasion *in vitro***. (D) Transwell assay of AN3CA cells transfected with SRC siRNA or NCs. ^*^P < 0.05.

